# Colchicine use might be associated with lower mortality in COVID‐19 patients: A meta‐analysis

**DOI:** 10.1111/eci.13645

**Published:** 2021-07-18

**Authors:** Mohamed Nabil Elshafei, Ahmed El‐Bardissy, Ahmed Khalil, Mohammed Danjuma, Mahmood Mubasher, Ibrahim Y. Abubeker, Mouhand F. H. Mohamed

**Affiliations:** ^1^ Clinical Pharmacy Department Hamad Medical Corporation Doha Qatar; ^2^ College of Medicine Qatar University Doha Qatar; ^3^ Department of Medicine Hamad Medical Corporation Doha Qatar; ^4^ Department of Internal Medicine Rochester Regional Health Unity Hospital of Rochester Rochester NY USA; ^5^ Alpert Medical School Brown University Providence RI USA

**Keywords:** colchicine, coronavirus disease 2019, COVID‐19, mortality, SARS‐CoV‐2

## Abstract

**Background:**

Colchicine was recently repurposed for the management of coronavirus disease 2019 (COVID‐19). This rapid review and meta‐analysis aimed to assess colchicine's impact on mortality outcomes in COVID‐19 patients.

**Materials and Methods:**

We systematically searched PubMed, EMBASE, Google Scholar since their inception till 25/03/2021 for observational or controlled studies that reported mortality as an outcome. The mortality odd ratios were generated with their corresponding 95% confidence intervals utilizing the random‐effects model.

**Results:**

Nine studies comprising 5522 patients met our inclusion criteria. Our meta‐analysis revealed significantly lower mortality in the colchicine group (OR 0.35, 95% CI 0.25‐0.48, I2 0%) compared with controls. A subgroup analysis limited to hospitalized patients (OR 0.35, 95% CI 0.25‐0.50, I2 0%) revealed similarly lower mortality in the colchicine group.

**Conclusions:**

This meta‐analysis suggests a mortality benefit with colchicine when used in the treatment of COVID‐19 patients. The majority of included studies were observational; thus, the findings of this review need to be further supported by the results of ongoing trials.

We read with great interest the recently published meta‐analysis by Aimo et al^1^ in the European Journal of Clinical Investigation. The analysis encompassing over 5000 patients' data revealed a significant reduction in adverse cardiovascular events in patients with chronic coronary syndrome receiving colchicine vs. control. These results are promising and suggest a potential role for colchicine in treating thrombogenic conditions. Colchicine is an ancient anti‐inflammatory agent with an established safety profile. It inhibits various inflammatory pathways, including neutrophils adhesion, inflammasome activation, microtubule formation, neutrophil extracellular traps (NETs) essential in the severe acute respiratory syndrome coronavirus 2 (SARS‐CoV‐2) pathogenesis.^2^, ^3^ Coronavirus disease 2019 (COVID‐19) is thought to be associated with an exaggerated inflammatory response and thrombogenicity.^4^ Thus, studies tested repurposing this medication in the treatment of COVID‐19 and yielded promising results.^5^, ^6^

We performed a rapid systematic review and meta‐analysis to examine the mortality effect in patients with COVID‐19 receiving colchicine vs. control. We followed our previously published protocol; however, we decided to accept observational studies for this rapid review due to data scarcity.^7^ We comprehensively searched PubMed, EMBASE, Google Scholar since their inception till 25/03/2021 for observational or controlled studies that reported mortality as an outcome. On screening, we limited the inclusion to articles written in the English language. We generated the mortality odds ratio with a 95% confidence interval utilizing the random effects model. We performed a subgroup analysis to examine the effect in hospitalized patients, also another analysis limited to peer‐reviewed publications. We generated a funnel plot to ascertain publication bias, and we performed a sensitivity analysis to check the results' consistency. MetaXl software was used for statistical analysis.

Nine studies comprising 5522 patients met our inclusion criteria comparing colchicine with control in the treatment of COVID‐19. Hence, they were included in the quantitative analysis (Table 1). Three of the studies were randomized controlled trials^3^, ^5^, ^8^: one was quasi‐experimental,^9^ and the remaining were observational.^2^, ^6^, ^10^, ^11^, ^12^ The only included nonpeer‐reviewed publication by Tardif et al^8^ accounted for the majority of included cases (4488 patients) and consisted of nonhospitalized patients. Patients in the intervention group received colchicine in different dosage regimens and were followed up to 30 days. All studies revealed numerically reduced mortality associated with colchicine use, albeit statistically insignificant in a few instances. The quality of most included studies was moderate. Our meta‐analysis revealed significantly lower mortality in the colchicine group (OR 0.35, 95% CI 0.25‐0.48, *I* ^2^ 0%) (Figure 1). A subgroup analysis limited to 902 hospitalized patients of which 433 received colchicine (OR 0.35, 95% CI 0.25‐0.50, *I* ^2^ 0%)^2^, ^3^, ^5^, ^6^, ^10^ and to peer‐reviewed publications including total of 1034 patients (OR 0.33, 95% CI 0.24‐0.47, *I* ^2^ 0%)^2^, ^3^, ^5^, ^6^, ^10^, ^11^ revealed similarly lower mortality in the colchicine group. The exclusion of constituent studies did not affect the results' consistency. There was no evidence of heterogeneity as depicted an *I* ^2^ of 0%. Moreover, sensitivity analysis, including two studies that we have excluded (studied colchicine in a poorly controlled manner), revealed a consistent effect on mortality (OR 0.43, 95% CI 0.31‐0.58, *I* ^2^ 13%).^13^, ^14^ The funnel plot revealed asymmetry suggesting a possibility of a publication bias.

**TABLE 1:** Characteristics of included studies

Study author (country)	Design	Median age (male%) colchicine/ Median age (male%) control	Patient setting	Intervention	Follow‐up duration	Primary outcomes	Mechanical ventilation n/N (%)	Mortality n/N (%)
Deftereos et al 2020^5^ (Greece)	RCT	63 (56.4%)/ 65 (60%)	Inpatient	Colchicine 1.5 mg × 1 dose >0.5 mg after 60 min >maintenance of 0.5 mg BID up to 3 wk	Hospital discharge or up to 21 d	1. Time to deterioration. 2. Maximum high‐sensitivity cardiac troponin level 3. Time for C‐reactive protein to reach more than 3 times the upper reference limit.	Colchicine 1/55 (1.8%) Control 5/50 (10%)	Colchicine 1/55 (1.8%) Control 4/50 (8%)
Scarsi et al 2020^6^ (Italy)	Prospective cohort study	69.3 (63%)/ 70.5 (64%)	Inpatient	Colchicine 1mg OD, reduced to 0.5 mg/d if severe diarrhoea (duration NS)	Recruitment March 5‐April 5, 2020 and patients followed till April 16 The study reported 21 d of survival.	Survival rate	NS	Colchicine 20/122 (16%) Control 52/140 (37.1%)
Sandhu et al 2020^10^ (USA)	Case‐control study	70 (64.2%)/ 65 (55.6%)	Inpatient	Colchicine 0.6 mg BID × 3 d >0.6 mg OD up to 12 d	Follow‐up period NS	1. Hospitalization days 2. Mortality 3. Mechanical ventilation 4. Discharge rate	Colchicine 28/53 (52.8%) Control 106/144 (73.6%)	Colchicine 26/53 (49%) Control 105/144 (72.9%)
Brunetti et al 2020^2^ (USA)	Prospective cohort study	61.2 (68.3%)/ 63 (70.7%)	Inpatient (severe COVID‐19)	Colchicine 1.2 mg × 1 dose >Maintenance 0.6 mg BID (duration NS)	Up to 28 d	In‐hospital mortality within 28 d	NS	Colchicine 3/33 (9.1%) Control: 11/33 (33.3%)
Lopes et al 2020^17^ (Brazil)	RCT	48 (52.9%)/ 53.5 (27.8%)	Inpatient (moderate to severe COVID‐19)	Colchicine 0.5 mg TID × 5 d >0.5 BID ×5 d	‐Recruitment April 11‐July 6, 2020 (follow‐up period NS)	1. Time to need for supplemental oxygen; 2. Time to hospitalization. 3. Need for admission and length of stay in ICU 4. Death rate	NS	Colchicine: 0/36 (0%) Control: 2/36 (6%)
Tardif et al 2020^8^ (Canada)	RCT	54.4(44.6%) 54.9(47.5%)	Outpatient (mild to moderate COVID‐19)	0.5 mg BID × 3 d >OD × 27 d	Up to 30 d	Composite of death or hospitalization due to COVID‐19 infection	Colchicine: 11/2235 (0.5%) Control 21/2253 (0.9%)	Colchicine: 5/2235 (0.2%) Control: 9/2253 (0.4%)
Manenti et al 2021^11^ (Italy)	Retrospective cohort	60.5 (72.9%)/ 62.5 (69%)	Inpatient and outpatient	1 mg OD till clinical improvement (up to 21 d)	Up to 21 d	1. Differences in mortality 2. Clinical improvement 3. Inflammatory markers	NS	Colchicine 5/66 (7.5%) Control: 19/66 (28.5%)
García‐Posada et al^12^ (Columbia)	Retrospective cohort	60 (61%) (overall, NS for each group separately)	Inpatient (moderate to severe COVID‐19)	Dose and duration NS	Follow‐up period NS	Differences in mortality between treatment groups	NS	Colchicine 56/113 (49.5%) Control: 29/44 (65.9%)
COLORIT 2021^9^ (Russia)	Quasi‐randomized trial	61.9(66.7%)/ 59.9(72.7%)	Inpatient (moderate to severe COVID‐19)	1 mg OD × 1‐3 d >0.5 mg OD (up to 14 d)	Up to discharge or 12 d	Changes in the SHOCS‐COVID score.	NS	Colchicine 0 (0%) Control: 2 (9.09%)

Abbreviations: >, followed by; BID, twice daily; COVID‐19, coronavirus disease 2019; NS, Nonspecified; OD, once daily; RCT, randomized clinical trial*SHOCS*‐COVID, Symptomatic Hospital and Outpatient Clinical Scale for *COVID*‐*19*.

**FIGURE 1:**
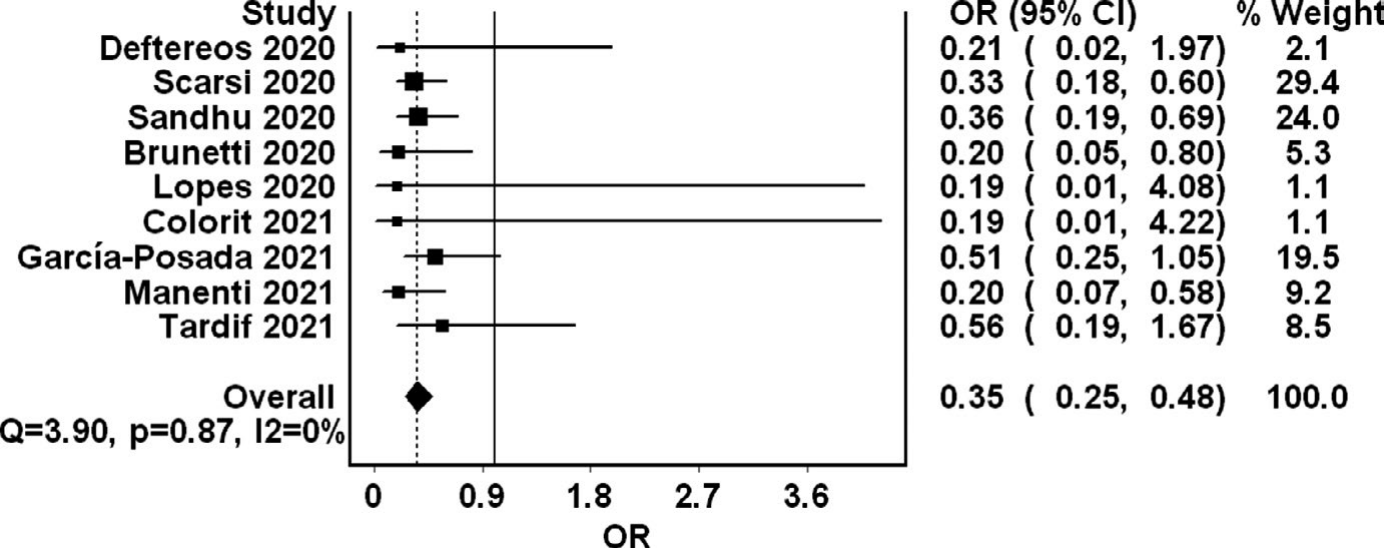
Forest plot summarizing the pooled mortality odds in COVID‐19 patients receiving colchicine compared to controls

Our analysis revealed lower mortality associated with colchicine use. Significant immunosuppressed status and predisposition to infections seen with other immunomodulators are not commonly seen with colchicine.^5^, ^15^ This may have contributed to the mortality benefit seen with colchicine and not with many other immunomodulators. Moreover, endothelial dysfunction and vascular inflammation play an integral role in SARS‐CoV‐2 pathogenesis. This has led to a significant risk of thrombosis in this patient cohort.^4^ In an autopsy study by Wichmann et al,^16^ deep venous thromboses were found in 58%, and pulmonary embolism was the direct cause of mortality in a third of COVID‐19 patients. Deftereos and Sandhu et al found a lower rise of d‐dimers in COVID‐19 patients receiving colchicine compared to the standard of care.^5^, ^10^ These observations may suggest a potential role of colchicine in mitigating COVID‐19 thrombogenicity, thereby preventing fatal thrombotic events in COVID‐19 patients. Nonetheless, d‐dimers reduction might be due to the anti‐inflammatory properties of colchicine and may not necessarily correlate with thrombotic events. To further explore this effect, prospective‐related studies should account for venous and arterial thrombotic events as secondary outcomes and correct for these when ascertaining mortality outcomes.

Our review has limitations, including the observational nature of the majority of the included studies, varying severity of included patients, varying follow‐up durations, different dosages and durations of colchicine used in the individual studies, mortality was a secondary outcome in most studies and the inability to rule out a publication bias. Moreover, the large reliance on the preprint of Tardiff et al’ study is another limitation. All these may have affected the analysis conclusion. Nonetheless, the review encompassed a large number of patients, and the effect was consistent across constituent studies.

In summary, results from this meta‐analysis suggest lower mortality in COVID‐19 patients treated with colchicine. Colchicine is a low‐cost, widely available drug with a known safety profile. Thus, it may play a fundamental role in preventing COVID‐19‐associated dysregulated inflammatory response and, perhaps, its related thrombogenicity without causing significant immunosuppression. These findings are to be further supported by the results of ongoing RCTs.

## CONFLICT OF INTEREST

None declared by all authors.

## AUTHOR CONTRIBUTIONS

Contribution: MFHM, MNE and AE. Conceptualization: AE, MFHM and MNE. Methodology: MFHM and MNE. Data analysis: MFHM and MNE. Data Curation: AE, MNE and MFHM. Writing ‐ Original Draft: MNE, AE and MFHM. Writing ‐ Review & Editing: MFHM, MD, MM, IYA and AK.

## ETHICAL APPROVAL

No ethical approval is necessary as this was a secondary synthesis of published articles.

## References

[1] Aimo A, Pascual Figal DA, Bayes‐Genis A, Emdin M, Georgiopoulos G. Effect of low‐dose colchicine in acute and chronic coronary syndromes: a systematic review and meta‐analysis. Eur J Clin Invest. 2021;51(4):e13464. 10.1111/eci.13464 33251579

[2] Brunetti L, Diawara O, Tsai A, et al. Colchicine to Weather the Cytokine Storm in Hospitalized Patients with COVID‐19. J Clin Med. 2020;9(9):2961. 10.3390/jcm9092961 PMC7565543 32937800

[3] Lopes MI, Bonjorno LP, Giannini MC, et al. Beneficial effects of colchicine for moderate to severe COVID‐19: a randomised, double‐blinded, placebo‐controlled clinical trial. RMD Open. 2021;7(1):e001455. 10.1136/rmdopen-2020-001455 33542047 PMC7868202

[4] Mohamed MFH, Al‐Shokri SD, Shunnar KM, et al. Prevalence of Venous Thromboembolism in Critically Ill COVID‐19 Patients: Systematic Review and Meta‐Analysis. Front Cardiovasc Med. 2021;7:598846. 10.3389/fcvm.2020.598846 33585578 PMC7874113

[5] Deftereos SG, Giannopoulos G, Vrachatis DA, et al. Effect of colchicine vs standard care on cardiac and inflammatory biomarkers and clinical outcomes in patients hospitalized with coronavirus disease 2019: the GRECCO‐19 randomized clinical trial. JAMA Netw open. 2020;3(6):e2013136. 10.1001/jamanetworkopen.2020.13136 32579195 PMC7315286

[6] Scarsi M, Piantoni S, Colombo E, et al. Association between treatment with colchicine and improved survival in a single‐centre cohort of adult hospitalised patients with COVID‐19 pneumonia and acute respiratory distress syndrome. Ann Rheum Dis. 2020;79(10):1286‐1289. 10.1136/annrheumdis-2020-217712 32732245 PMC7509521

[7] Elshafei MN, Khalil A, El‐Bardissy A, Danjuma M, Ahmed MB, Mohamed MFH. The efficacy of colchicine in the management of coronavirus disease 2019: A protocol for systematic review and meta‐analysis. Medicine. 2020;99(36):e21911. 10.1097/MD.0000000000021911 32899023 PMC7478773

[8] Tardif J‐C, Bouabdallaoui N, L’Allier PL, et al. Efficacy of colchicine in non‐hospitalized patients with COVID‐19. medRxiv. 2021. 10.1101/2021.01.26.21250494

[9] Mareev VY, Orlova YA, Plisyk AG, et al. Proactive anti‐inflammatory therapy with colchicine in the treatment of advanced stages of new coronavirus infection. The first results of the COLORIT study. Kardiologiia. 2021;61(2):15‐27. 10.18087/cardio.2021.2.n1560 33734043

[10] Sandhu T, Tieng A, Chilimuri S, Franchin G. A case control study to evaluate the impact of colchicine on patients admitted to the hospital with moderate to severe covid‐19 infection. Can J Infect Dis Med Microbiol. 2020;2020: 10.1155/2020/8865954 PMC7588830 33133323

[11] Manenti L, Maggiore U, Fiaccadori E, et al. Reduced mortality in COVID‐19 patients treated with colchicine: Results from a retrospective, observational study. Cannatà A, ed. PLoS ONE. 2021;16(3):e0248276. 10.1371/journal.pone.0248276 33760858 PMC7990208

[12] García‐Posada M, Aruachan‐Vesga S, Mestra D, et al. Clinical outcomes of patients hospitalized for COVID‐19 and evidence‐based on the pharmacological management reduce mortality in a region of the Colombian Caribbean. J Infect Public Health. 2021;14(6):696‐701. 10.1016/j.jiph.2021.02.013 34020208 PMC8101281

[13] Alejandro Pinzón M, Medellin Doris Cardona Arango C, Felipe Betancur J, et al. Clinical Outcome of Patients with COVID‐19 Pneumonia Treated with Corticosteroids and Colchicine in Colombia. Published online October 23, 2020. 10.21203/rs.3.rs-94922/v1 PMC8438650 34521428

[14] Mahale N, Rajhans P, Godavarthy P, et al. A Retrospective Observational Study of Hypoxic COVID‐19 Patients Treated with Immunomodulatory Drugs in a Tertiary Care Hospital. Indian J Crit Care Med. 2020;24(11):1020‐1027. 10.5005/jp-journals-10071-23599 33384506 PMC7751026

[15] Kimmig LM, Wu D, Gold M, et al. IL‐6 Inhibition in Critically Ill COVID‐19 Patients Is Associated With Increased Secondary Infections. Front Med (Lausanne). 2020;7 10.3389/fmed.2020.583897 PMC7655919 33195334

[16] Wichmann D, Sperhake J‐P, Lütgehetmann M, et al. Autopsy findings and venous thromboembolism in patients with COVID‐19. Ann Int Med. 2020;173(4):268–277. 10.7326/M20-2003 32374815 PMC7240772

[17] Lopes MI, Bonjorno LP, Giannini MC, et al. Beneficial effects of colchicine for moderate to severe COVID‐19: a randomised, double‐blinded, placebo‐controlled clinical trial. RMD Open. 2021;7 (1):e001455. 10.1136/rmdopen-2020-001455 33542047 PMC7868202

